# Impact of physical exercise on health and social interaction in older adults: a meta-analysis

**DOI:** 10.3389/fpubh.2025.1634313

**Published:** 2025-09-18

**Authors:** Meng Tao, Shuang Li, Luyao Li, Yuanyuan Cao, Jie Zhuang

**Affiliations:** ^1^School of Exercise and Health, Shanghai University of Sport, Shanghai, China; ^2^Taizhou Vocational College of Science and Technology, Taizhou, China

**Keywords:** physical exercise, physical health, mental health, social interaction, older adults, meta-analysis

## Abstract

**Background:**

This study aimed to evaluate the effect of physical exercise on the physical and mental health of older adults using meta-analysis and to analyze the moderating variables influencing this relationship. The goal was to provide a scientific basis for identifying effective exercise methods to enhance the physical and mental health of the older adults.

**Methods:**

A literature search was conducted in databases such as PubMed, Web of Science, Cochrane Library, and Google Scholar. Data were processed using Review Manager 5.4 and Stata 18.0 statistical software. Meta-analysis was performed using the random effects model, and relevant moderating variables were tested.

**Results:**

The study findings indicated that physical activity significantly improves the physical and mental health of older adults across various dimensions. Specifically, it showed a large effect size for enhancing social interactions (standardized mean difference (SMD) = 0.78, 95% confidence interval (CI) [0.14, 0.69], *P* = 0.0002), a medium effect size for improving physical health (SMD = 0.42, 95% CI [0.20, 0.48], *P* = 0.003), and a small effect size for improving mental health (SMD=0.34, 95% CI [0.37, 1.19], *P* < 0.001). All differences were statistically significant.

**Conclusion:**

Physical exercise has a positive impact on improving the physical and mental health of older adults, particularly in enhancing physical health, psychological well-being, and social interactions.

## Background

The accelerated global aging process has led to increasingly prominent health problems among the older adults. Chronic diseases and social isolation have significantly reduced their quality of life (1). According to the World Health Organization (WHO) projections, by 2050, individuals aged 65 and older will account for one-sixth of the global population, further intensifying the trend of aging (2). Consequently, the health and quality of life of older adults have become a central concern. However, the quality of life among the older adults is often compromised by chronic diseases, social isolation, and family loneliness. Particularly, during the COVID-19 pandemic and its aftermath, many older adults have exhibited heightened vulnerability to both physical and psychological stress (3). Therefore, a comprehensive study of the physical and mental health status of the older adults is crucial for enhancing their quality of life and well-being (4, 5).

Existing studies demonstrate that physical exercise, as an intervention strategy, can significantly improve the mental health of older adults, in addition to positively affecting metabolic syndrome-related diseases (6). Moderate aerobic exercises, such as walking, jogging, and Tai Chi, not only enhance cardiorespiratory fitness and improve blood circulation but also promote the release of “pleasure hormones”, such as endorphins, which help alleviate anxiety and depression (7). Group physical activities, including square dancing and group aerobics, offer opportunities for social interaction, helping older adults establish new interpersonal relationships, reduce loneliness, and improve overall social adaptability and well-being (8).

Physical exercise, as a potential intervention, has been proven to improve metabolic health and mental health. However, previous studies have obvious limitations: on the one hand, most meta-analyses focus on clinical patients or are based on survey data, lacking systematic analyses for non-diseased older adults people (9). On the other hand, existing research has insufficient exploration of key parameters such as intervention intensity and frequency, making it difficult to guide practice (10). In response, the present study systematically reviewed randomized controlled trials investigating the effects of physical activity on the physical and mental health of non-diseased older adults. It also summarized and analyzed the critical dimensions of health status (e.g., physical health, mental health, and social interaction) and core parameters of the independent variables, including types, intensity, duration, frequency, and intervention period. The aim was to provide valuable insights for future research on physical activity interventions and their effects on the physical and mental health of older adults.

## Methods

### Search strategy

Regarding the selection of languages for the literature, English-language studies were prioritized due to their widespread dissemination and accessibility. Articles published between 2010 and 2023 were searched in the PubMed, Web of Science, Cochrane Library, and Google Scholar databases for relevant topics, which provide access to a vast collection of high-quality research. This approach ensured the comprehensiveness and reliability of the included literature. Furthermore, English language studies generally undergo rigorous peer review, adhere to standardized study designs, and provide detailed and reliable data reporting, enhancing their suitability for meta-analysis. The search terms consisted of a combination of three components: (1) “physical exercise” OR “sport” OR “physical activity ” OR “fitness” OR “training” OR “exercise” OR “Physical training”; (2) “psychological stress” OR “psychological” OR “well-being” OR “mood” OR “psychologically” OR “relaxation” OR “stress reduction” OR “restoration” OR “emotional” OR “affective” OR “mental” OR “attention”; (3) “older adults” OR “aged” OR “older adults” OR “older individuals ” OR “older people” OR “senior citizens”. To ensure the validity and scientific rigor of the research, the study was first registered on the PROSPERO platform and received approval prior to its commencement (registration number: CRD42024553544).

### Inclusion and exclusion criteria

The inclusion criteria were as follows: (1) study types: randomized controlled trial (RCT) design; (2) study subjects: individuals aged over 60 years with no chronic diseases; (3) intervention method: the intervention group participated in at least one type of physical exercise, including specific details on time, frequency, intensity, and duration; (4) outcome indicators: at least one physical or mental health measure. The exclusion criteria were: (1) non-randomized controlled trials; (2) lack of physical activity intervention; (3) age outside the specified range; (4) missing relevant data; (5) duplicate publications. Due to insufficient quality control and the lack of data standardization, in order to ensure the rigor of the analysis, this study focuses on peer-reviewed RCT literature For articles where disagreement arise, this discussion should reference the original literature, adhere to the pre-established screening criteria, and consider the broader research context in the field. If consensus cannot be reached, a third researcher, an expert in the field of exercise and health promotion from Shanghai University of Sport, will be invited to arbitrate.

### Literature screening

**Figure 1:**
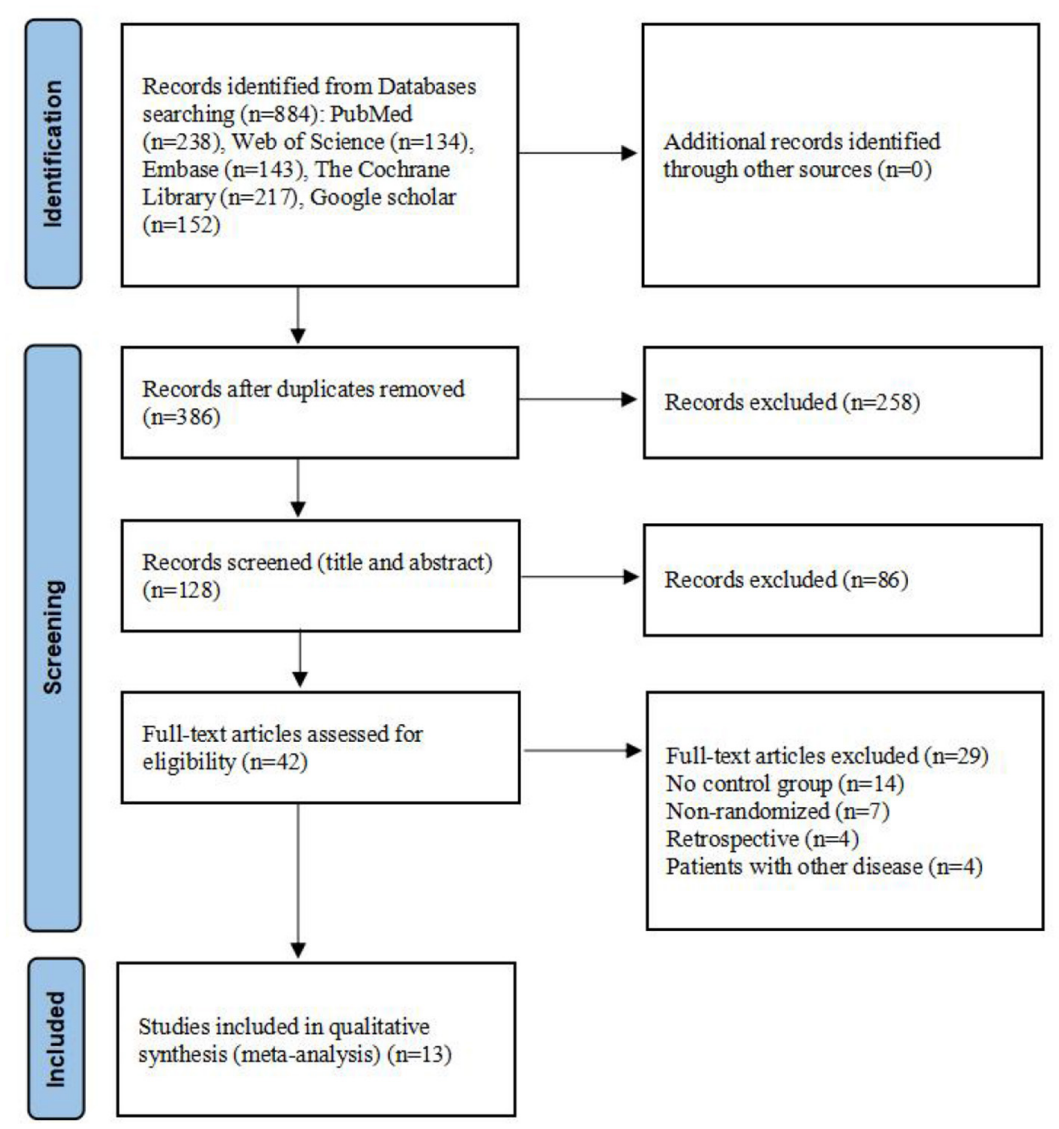
Flow chart of literature retrieval.

A total of 884 articles were identified from the database. The search and evaluation process was carried out by two independent investigators, who screened the articles based on predefined inclusion and exclusion criteria. Ultimately, 13 articles and 15 analyses were included in the meta-analysis, because some of these studies included multiple groups. The studies by Alida (2019a, 2019b) indicate that the author utilized two types of interventions. Similarly, the studies by Chaiya (2017a, 2017b) also reflect the use of two intervention types. Tiia (2017a, 2017b, 2017c) adopted three intervention types in their studies. Flavia (2015a, 2015b) and Ulku (2018a, 2018b) both employed two types of interventions in their respective studies. The PRISMA flowchart outlining the retrieval process is shown in Figure 1.

### Quality assessment

**Figure 2:**
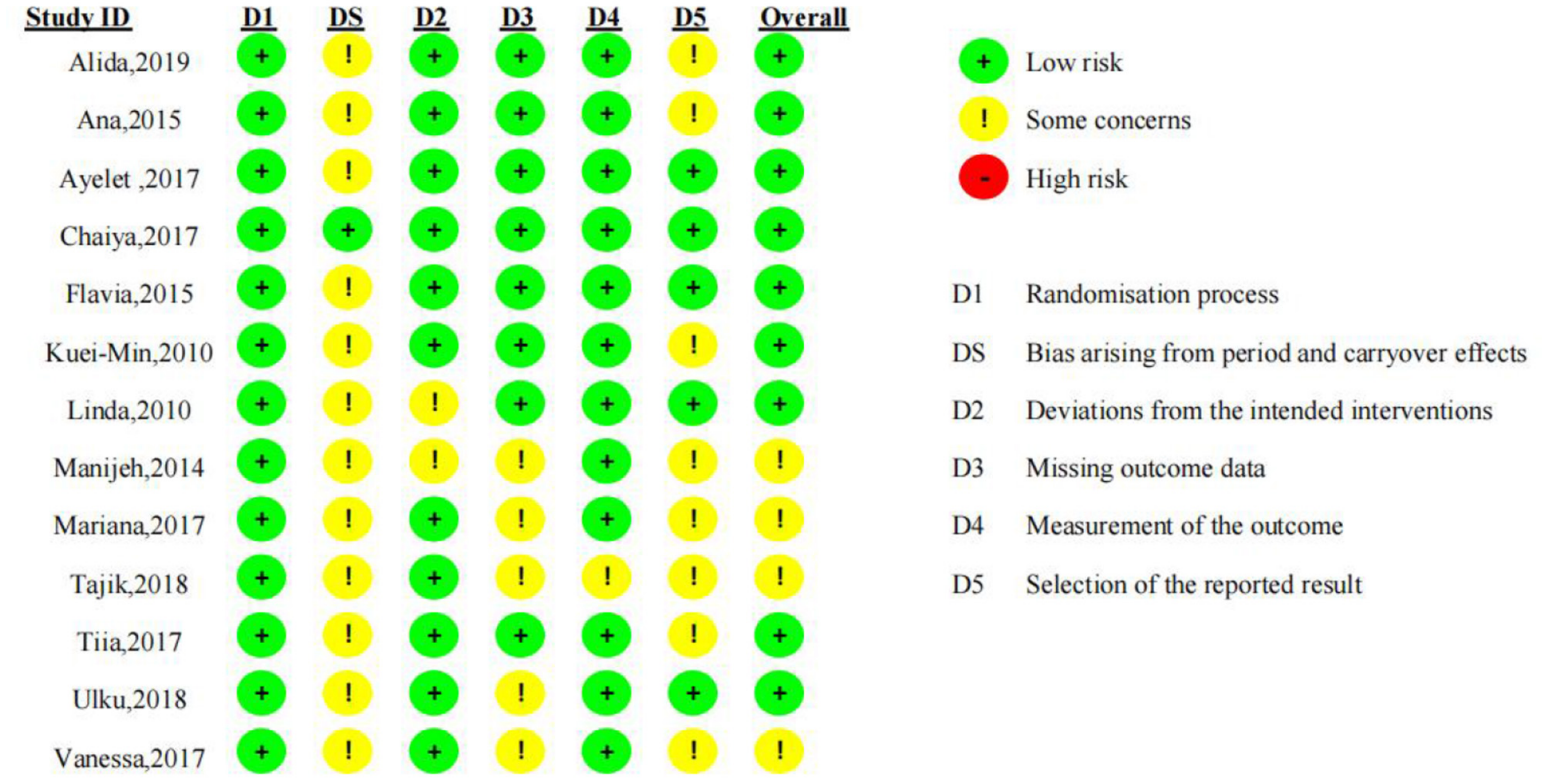
Risk of bias of the included studies.

The quality of the 15 included RCTs was assessed using the Cochrane Risk of Bias 2.0 tool (11), and Review Manager 5.4. Detailed results of the literature quality evaluation are presented in Figure 2, the evaluation focused on the following aspects: (1) selection bias: whether the method of random sequence generation was used; (2) allocation concealment: whether the allocation was effectively concealed; (3) blinding: whether the subjects or investigators were blinded; (4) data integrity: whether missing data was adequately reported and intention-to-treat analysis was applied; (5) selective reporting: whether there was any selective reporting of outcomes; (6) other biases: whether other factors contributed to potential bias (10). Jadad scale was used to evaluate literature quality (12). 1. Random sequence generation: appropriate (2 points), unclear (1 point), inappropriate (0 points); 2. Allocation concealment: appropriate (2 points), unclear (1 point), inappropriate (0 points); 3. Blinding: appropriate (2 points), unclear (1 point), inappropriate (0 points); 4. Withdrawals and dropouts: described with numbers and reasons (1 point), not described (0 points). Based on the total score, studies were classified as low-quality (1–3 points) or high-quality (4–7 points). In cases of disagreement regarding the quality assessment, a third researcher conducted the evaluation. The quality assessment of the included studies is shown in Table 1.

**Table 1:** Quality assessment of included studies.

**Author**	**1**	**2**	**3**	**4**	**Score**	**Quality**
Alida (2019) (13)	2	0	0	0	2	Low
Ana (2015) (44)	2	2	0	1	5	High
Ayelet (2017)(19)	2	1	2	0	5	High
Chaiya, 2017 (14)	1	1	1	1	4	High
Flavia, 2015 (15)	2	1	1	0	4	High
Kuei-Min, 2010 (27)	2	1	1	1	5	High
Linda, 2010 (16)	2	1	1	0	4	High
Manijeh, 2014 (17)	1	1	1	1	4	High
Mariana, 2017 (18)	2	1	1	1	5	High
Tajik, 2018 (20)	1	1	1	0	3	Low
Tiia, 2017 (21)	2	1	1	1	5	High
Ulku, 2018 (22)	2	1	1	0	4	High
Vanessa, 2017 (23)	1	1	1	0	3	Low

1. Random sequence generation: appropriate (2 points), unclear (1 point), inappropriate (0 points); 2. allocation concealment: appropriate (2 points), unclear (1 point), inappropriate (0 points); 3. blinding: appropriate (2 points), unclear (1 point), inappropriate (0 points); 4. withdrawals and dropouts: described with numbers and reasons (1 point), not described (0 points). Based on the total score, studies were classified as low-quality (1–3 points) or high-quality (4–7 points).

### Data extraction

Two researchers independently extracted data from the included studies, including the source of the literature, publication year, sample size, participants' characteristics, age, intervention type, duration, trial design, study period, frequency, type, and intensity. The standards for measuring intervention intensity varied across studies. In this analysis, relevant data such as maximum oxygen uptake (VO_2_max), reserve heart rate (HRR) (13, 14), maximum heart rate (HRmax), reserve oxygen uptake (VO_2_R) (15–18), and the Rate of Perceived Exertion (RPE) (15, 16, 19, 20) subjective intensity scale in resistance training were selected as benchmarks. Several studies on resistance training have utilized the repetition maximum (RM) as a method for assessing intensity (21–23). For studies that did not report specific measures or methods of intensity, we estimated intensity based on the Older Adult Compendium of Physical Activities (24). The estimated strength based on the manual may have errors, but the stability of the results has been verified through sensitivity analysis in the subsequent analysis. The measurement standards for different exercise intensities are provided in Table 2. In cases of discrepancies in data extraction, a third-party member discussed and resolved the inconsistencies.

**Table 2:** Common method of measuring exercise intensity.

**Intensity class**	**%HRR, %VO_2_R**	**%HR_max_**	**%VO_2max_**	**RPE (0–10)**
Low intensity	< 39	< 63	< 37	< 34
Moderate intensity	40–59	64–76	46–63	5–6
High intensity	>60	>77	>64	>7

HRR, reserve heart rate; VO_2_R, reserve oxygen uptake; HR_max_, maximum heart rate; VO_2max_, maximal oxygen uptake; RPE, rate of perceived exertion.

### Evaluation of evidence level

Evidence quality was assessed using the grading of recommendations assessment, development and evaluation (GRADE), which classifies evidence as high, moderate, low, or very low quality. The evaluation criteria included risk of bias, inconsistency, indirectness, imprecision, and publication bias. Two researchers independently performed the GRADE assessments.

### Statistical analysis

This study examined three dimensions of health: physical health, mental health, and social interaction. Statistical analysis was performed using Review Manager 5.4 software and Stata 18.0 software. All outcome indicators were continuous variables. For outcomes measured using the same method and units, the mean difference (MD) and 95% confidence interval (CI) were used as effect size indicators. For outcomes with different measurement methods or units, the standardized mean difference (SMD) and 95% CI were used instead. The *X*^2^ test was employed to assess heterogeneity among the included studies (α = 0.1). If *P* ≥ 0.1 and *I*^2^ < 50%, the heterogeneity was considered low, and a fixed-effect model (FEM) was used for analysis. If *P* < 0.1 and *I*^2^ ≥ 50%, the heterogeneity was considered high, and a random-effects model (REM) was used for analysis. Stata 18.0 software was used to conduct sensitivity analysis and generate funnel plots. Additionally, publication bias tests were performed on the studies included in each dimension, so we refer to Higgins abd Thompson (25) methodology for addressing heterogeneity. Given the inherent subjectivity of funnel plot interpretation, both the Egger and Begg tests were employed to more accurately assess publication bias. A *P*-value < 0.05 was considered indicative of publication bias.

## Results

### Research characteristics

A total of 13 randomized controlled trials were included in this meta-analysis, with specific details provided in Table 3. The analysis included 1,176 healthy older adults, aged 60 to 96 years. Physical and mental health were assessed using various measurement tools, including the Subjective Life Aatisfaction (SLS), Taiwanese Depression Questionnaire (TDQ), Satisfaction with the Nursing Home Instrument (SNHI), Social Support Questionnaire-Short Form (SSQ-6), Short Form-12 Physical And Mental Sub-Scales (SF-12), 36-Item Of Short-Form Health Survey (SF-36), State Self-Esteem Scale (SSES), Who Quality Of Life Instrument-Older Adults (WHOQOL-OLD), Geriatric Depression Scale (GDS), Leiden Padua Quality Of Life Questionnaire (LEIPAD), Beck Depression Inventory II (BDI-II), Portuguese Version of WHO-5 (WHO-5) and other questionnaires. The participants were primarily selected from community settings and nursing institutions, with a focus on older adults individuals in good health, free from chronic or infectious diseases. Intervention methods included tai chi, yoga, stationary cycling, gymnastics, dance, and other training modalities. The interventions lasted between 30 and 90 min per session, occurred 2–3 times per week, and spanned 8 to 32 weeks.

**Table 3:** Basic information table of included literatures.

**Author**	***N***	**Age**	**Types**	**Measure**	**Intensity**	**Time**	**Frequency**	**Period**	**Design**	**Subjects**	**Scales**
Alida, 2019a (13)	39	≥60	A-T	Dance training	H-I	60 min	3 times/week	12 weeks	RCT	C15, E12	QoL
Alida, 2019b (13)	41	≥60	A-T	Aerobic training	L-I	60 min	3 times/week	12 weeks	RCT	C14, E12	QoL
Ana, 2015 (44)	57	65–80	A-T	Dance	L-I	50 min	3 times/week	24 weeks	RCT	C32, E25	SLS
Ayelet, 2017 (19)	27	64–72	A-T	Aerobic step	M-I	45 min	2 times/week	8 weeks	RCT	C13, E14	SF-36
Chaiya 2017a (14)	21	60–74	A-T	Yoga	L-I	90 min	2 times/week	28 weeks	RCT	C10, E11	SF-36; GDS
Chaiya 2017b (14)	19	60–74	A-T	Shadow boxing	L-I	90 min	2 times/week	12 weeks	RCT	C10, E9	SF-36; GDS
Flavia 2015a (15)	55	60–75	A-T	Aerobic running	M-I	60 min	3 times/week	32 weeks	RCT	C31, E24	SF-36
Flavia 2015b (15)	55	60–75	R-T	Resistance training	M-I	60 min	3 times/week	32 weeks	RCT	C31, E19	SF-36
Kuei-Min, 2010 (27)	128	62–75	A-T	Yoga	L-I	70 min	3 times/week	24 weeks	RCT	C64, E62	TDQ
Linda, 2010 (16)	139	76–90	A-T	Shadow boxing	L-I	60 min	3 times/week	26 weeks	RCT	C73, E66	SSES, SF-12, SSQ6, SNHI
Manijeh, 2014 (17)	62	65–75	A-T	Aerobic running	M-I	30 min	3 times/week	6 weeks	RCT	C32, E30	LEIPAD
Mariana, 2017 (18)	25	≥60	A-T	Yoga	M-I	50 min	2 times/week	12 weeks	RCT	C10, E15	WHO-5
Tajik, 2018 (20)	132	≥60	A-T	Shadow boxing	L-I	30-40 min	3 times/week	8 weeks	RCT	C66, E66	LEIPAD
Tiia, 2017a (21)	52	65–75	R-T	Resistance training	H-I	60 min	1 times/week	12 weeks	RCT	C19, E24	WHOQOL-OLD, BDI-II
Tiia, 2017b (21)	52	65–75	R-T	Resistance training	H-I	60 min	2 times/week	12 weeks	RCT	C19, E25	WHOQOL-OLD, BDI-II
Tiia, 2017c (21)	53	65–75	R-T	Resistance training	H-I	60 min	3 times/week	12 weeks	RCT	C19, E27	WHOQOL-OLD, BDI-II
Ulku, 2018a (22)	32	69–96	R-T	Resistance training	H-I	40 min	3 times/week	8 weeks	RCT	C16, E16	WHOQOL-OLD, GDS-15
Ulku, 2018b (22)	32	69–96	R-T	Resistance training	L-I	40 min	3 times/week	8 weeks	RCT	C16, E16	WHOQOL-OLD, GDS-15
Vanessa, 2017 (23)	61	64	A-T	Pilates	L-I	60 min	2 times/week	16 weeks	RCT	C30, E31	SLS

A-T, aerobic training; R-T, resistance training; L-I, low intensity; M-I, moderate intensity; H-I, high intensity; C, control group; E, experiment group; QoL, quality of life; SLS, subjective life satisfaction; TDQ, Taiwanese depression questionnaire; SNHI, satisfaction with the nursing home instrument; SSQ6, social support questionnaire-short form; SF-12, short form-12 physical and mental sub-scales; SF-36, 36-item of short-form health survey; SSES, state self-esteem scale; WHOQOL-OLD, WHO quality of life instrument-older adults; GDS, geriatric depression scale; LEIPAD, Leiden Padua quality of life questionnaire; BDI-II, Beck depression inventory II; WHO-5, Portuguese version of WHO-5.

### Dimensions of health and social interaction

Each dimension of physical and mental health is relatively independent, with distinct influencing factors. To enhance the accuracy of the effect tests, this study first examines each dimension of physical and mental health separately. The three dimensions analyzed in this study—physical health, mental health, and social interaction—comprised 58 effect sizes from 13 studies and 15 analyses. Because some of these studies included multiple groups, which were included in the meta-analysis, as shown in Table 4.

**Table 4:** Physical exercise and meta-analysis of each dimension.

**Indicator**	***n***	**Heterogeneity test**			**SMD and 95% CI**	**Two-tailed test**	
		*X* ^2^	***P***	*I* ^2^		***Z***	***P***
Physical health	15	48.72	< 0.001	71%	0.42 [0.14, 0.69]	2.93	0.003
Mental health	15	22.16	0.09	37%	0.34 [0.20, 0.48]	4.69	< 0.001
Social interaction	15	103.25	< 0.001	86%	0.78 [0.37, 1.19]	3.71	0.0002

### Assessment of evidence quality

The quality of evidence for the included studies was graded based on the GRADE (Grading of Recommendations Assessment, Development, and Evaluation) system, with evaluation dimensions including risk of bias, inconsistency, indirectness, imprecision, and publication bias. Results are presented in Table 5.

**Table 5:** Evaluation results of evidence quality in each dimension.

**Indicator**	**Risk of bias**	**Inconsistency**	**Indirectness**	**Imprecision**	**Publication bias**	**Final quality**
Physical health	Moderate	High	Low	Low	Applicable	Moderate
Mental health	Low	Low	Low	Low	Applicable	High
Social interaction	Moderate	High	Low	Low	Not applicable	Low

### Physical health

**Figure 3:**
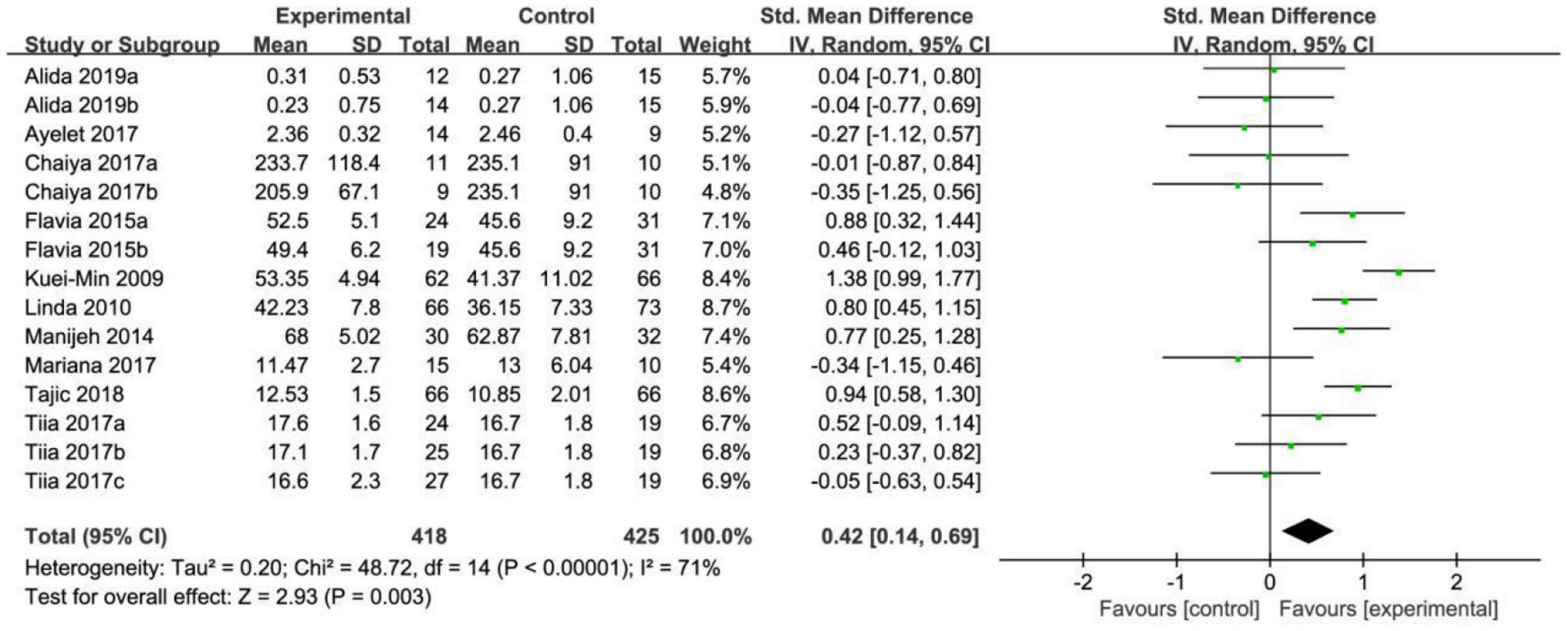
Forest map of the effects of physical exercise on physical health.

In the analysis of physical health, a total of 15 effect sizes were included in the meta-analysis. The heterogeneity test results indicated that χ^2^ = 48.72, *P* < 0.05, *I*^2^ = 71%, suggesting statistically significant differences and high heterogeneity among the studies. The test confirmed that the effect sizes did not originate from the same population, leading to the use of a random effects model (REM) for further analysis. The pooled effect size was SMD = 0.42, with a 95% confidence interval of (0.14, 0.69), and the test for the pooled effect size yielded *Z* = 2.93, *P* = 0.003. According to the analysis, older adults in the exercise intervention group showed significantly better physical health compared to those in the control group, as illustrated in Figure 3.

### Mental health

**Figure 4:**
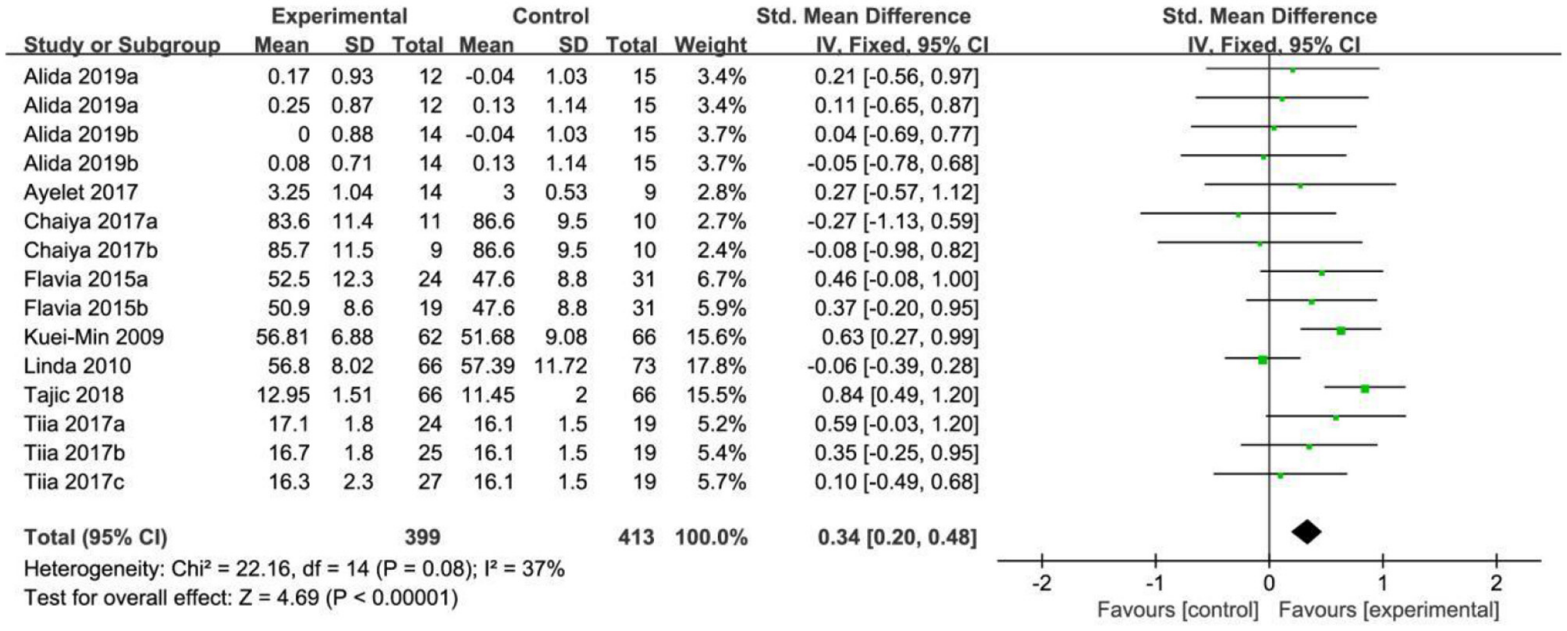
Forest map of the effects of physical exercise on mental health.

In the analysis of mental health, a total of 15 effect sizes were included in the meta-analysis. The heterogeneity test results indicated that *X*^2^ = 22.16, *P* = 0.08, *I*^2^ = 37%, suggesting low heterogeneity among the studies and statistically significant differences. The test results also showed that the selected effect sizes were generally consistent, prompting the use of a fixed effect model (FEM) for analysis. The combined effect size was SMD = 0.34, with a 95% confidence interval of (0.20, 0.48), and the combined effect size test yielded *Z* = 4.69, *P* < 0.001. According to the analysis, older adults in the exercise intervention group showed better mental health than those in the control group, as illustrated in Figure 4.

### Social interaction

**Figure 5:**
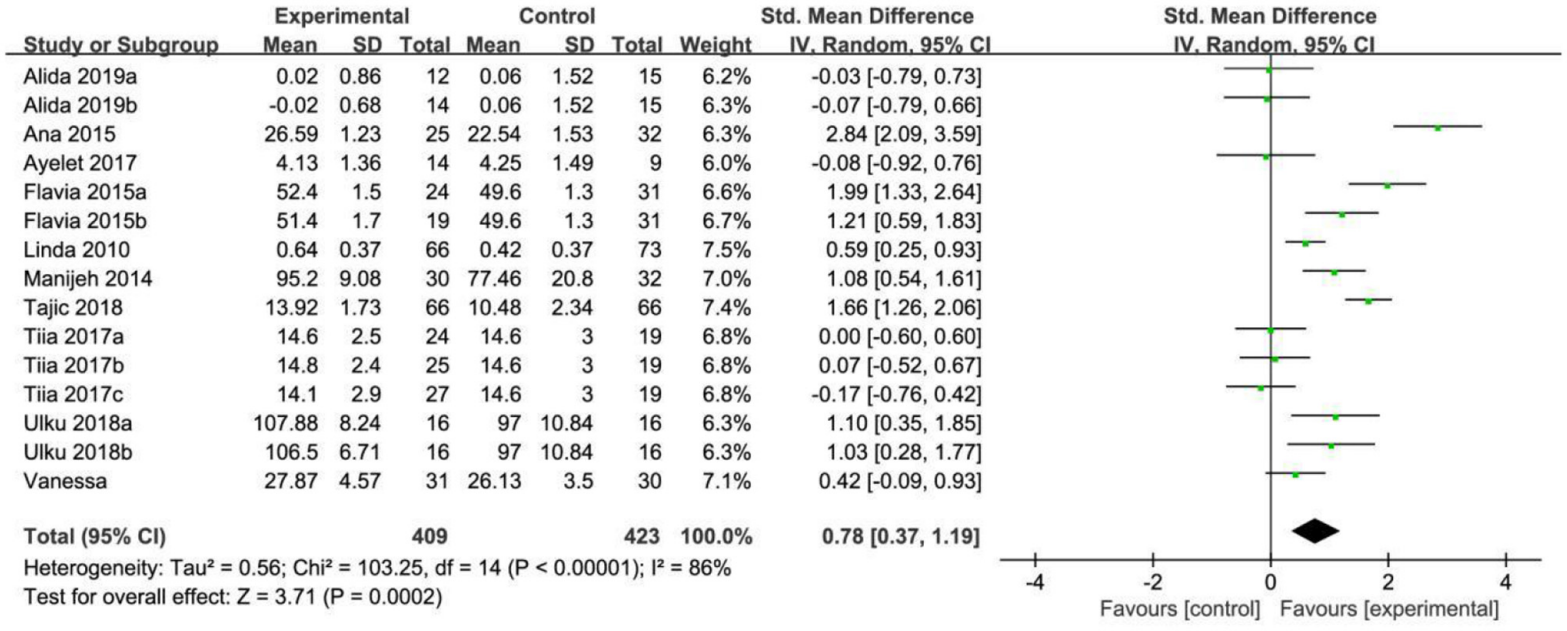
Forest map of the effects of physical exercise on social interaction.

In the analysis of social interaction, a total of 15 effect sizes were included in the meta-analysis. The heterogeneity test results indicated significant differences (*X*^2^ = 103.25, *P* < 0.001, *I*^2^ = 86%), suggesting a high degree of heterogeneity among the studies. The test confirmed that the effect sizes did not originate from the same population, so a random effects model (REM) was used for further analysis. The combined effect size was SMD = 0.78, with a 95% confidence interval of (0.37, 1.19), and the combined effect size test yielded Z = 3.71, *P* < 0.0002. According to the analysis, older adults in the intervention group showed significantly better social communication compared to the control group, as illustrated in Figure 5.

### Subgroup results

This study not only examines the three dimensions of physical health, mental health, and social interaction separately, but also investigates the moderating variables associated with physical and mental health. The subgroup classification was established based on the methodological framework proposed by and Arent et al. (26). Five regulating variables, including intervention type, duration, intensity, frequency, and period, were selected for effect testing, as shown in Table 6.

**Table 6:** Effect size tests for subgroup analysis.

**Indicator**	**Subgroup**	**Category**	***n***	**Heterogeneity test**			**SMD and 95%CI**	**Two-tailed test**	
				*X* ^2^	***P***	*I* ^2^		***Z***	***P***
Physical health	Type	A-T	11	40.53	< 0.001	75%	0.45 [0.10, 0.80]	2.54	0.01
		R-T	4	2.18	0.54	0%	0.29 [−0.01, 0.58]	1.89	0.06
	Intensity	L-I	6	23.82	0.0002	79%	0.58 [0.12, 1.04]	2.46	0.01
		M-I	5	10.25	0.04	61%	0.38 [−0.08, 0.84]	1.62	0.11
		H-I	4	10.81	0.01	72%	0.37 [−0.21, 0.94]	1.24	0.21
	Frequency	≥3 times/week	9	27.46	0.0006	71%	0.64 [0.33, 0.95]	4.06	< 0.001
		< 3 times/week	6	4.83	0.44	0%	0.06 [−0.24, 0.36]	0.40	0.69
	Time	≥60 min	11	35.52	0.0001	72%	0.42 [0.09, 0.75]	2.48	0.01
		< 60 min	4	13.14	0.004	77%	0.37 [−0.25, 0.98]	1.16	0.25
	Period	>12 weeks	6	19.58	< 0.001	74%	0.64 [0.19, 1.08]	2.82	0.005
		≤ 12 weeks	9	21.25	0.007	62%	0.27 [-0.06, 0.60]	1.59	0.11
Mental health	Type	A-T	11	20.87	0.02	52%	0.33 [0.17, 0.49]	4.09	< 0.001
		R-T	4	1.28	0.73	0%	0.35 [0.05, 0.64]	2.28	0.02
	Intensity	L-I	7	20.20	0.003	70%	0.34 [0.16, 0.52]	3.72	0.0002
		M-I	3	0.14	0.93	0%	0.39 [0.04, 0.75]	2.16	0.03
		H-I	5	1.59	0.81	0%	0.29 [−0.01, 0.58]	1.92	0.06
	Frequency	≥3 times/week	10	18.71	0.03	52%	0.35 [0.20, 0.51]	4.45	< 0.001
		< 3 times/week	5	3.19	0.53	0%	0.26 [−0.07, 0.59]	1.56	0.12
	Time	≥60 min	13	13.00	0.37	8%	0.24 [0.09, 0.40]	3.05	0.002
		< 60 min	2	1.48	0.22	33%	0.76 [0.43, 1.08]	4.51	0.0001
	Period	>12 weeks	6	10.38	0.07	52%	0.26 [0.06, 0.45]	2.58	0.01
		≤ 12 weeks	10	12.89	0.17	30%	0.38 [0.19, 0.58]	3.82	0.001
Social interaction	Type	A-T	10	74.56	< 0.001	88%	0.96 [0.44, 1.48]	3.60	0.003
		R-T	5	15.63	0.004	74%	0.41 [−0.14, 0.96]	1.45	0.15
	Intensity	L-I	6	52.38	< 0.001	90%	1.07 [0.37, 1.76]	3.01	0.003
		M-I	4	14.61	0.002	0%	1.08 [0.36,1.80]	2.95	0.003
		H-I	5	7.80	0.10	49%	0.16 [−0.24, 0.57]	0.79	0.43
	Frequency	≥3 times/week	11	78.84	< 0.001	87%	1.02 [0.53, 1.51]	4.07	0.001
		< 3 times/week	4	103.25	0.001	86%	0.16 [−0.14, 0.46]	3.71	0.0002
	Time	≥60 min	10	74.49	0.001	88%	0.67 [0.14, 1.21]	2.49	0.01
		< 60 min	5	32.35	< 0.001	88%	0.75 [−0.00, 1.50]	1.96	0.05
	Period	>12 weeks	10	74.49	< 0.001	88%	0.67 [0.14, 1.21]	2.49	0.01
		≤ 12 weeks	5	14.65	0.005	73%	1.02 [0.49, 1.56]	3.77	0.0002

A-T, aerobic training; R-T, resistance training; L-I, low intensity; M-I, moderate intensity; H-I, high intensity; C, control group; E, experiment group.

The results of the subgroup analysis on intervention methods revealed that aerobic training significantly improved physical health, mental health, and social interaction in older adults. Specifically, aerobic training had a large effect on physical health (SMD = 0.45, *P* = 0.01) and social interaction (SMD = 0.96, *P* = 0.003). However, its effect on mental health was comparatively smaller (SMD = 0.33, *P* < 0.001). In contrast, resistance training showed a significant effect on mental health (SMD = 0.35, *P* = 0.02), but no significant differences were observed in its impact on physical health or social interaction.

The results of the subgroup analysis of intervention intensity revealed that low-intensity physical exercise significantly improved physical health, mental health, and social interaction in older adults. Among these, low-intensity exercise had the largest effect on physical health (SMD = 0.58, *P* = 0.01) and social interaction (SMD = 1.07, *P* = 0.003), while the effect on mental health was relatively smaller (SMD = 0.34, *P* = 0.0002). Moderate-intensity exercise also showed significant effects on mental health (SMD = 0.39, *P* = 0.03) and social interaction (SMD = 1.08, *P* = 0.003), whereas high-intensity exercise showed no significant improvement in physical health, mental health, or social interaction.

The subgroup analysis of intervention frequency revealed that an intervention frequency of ≥3 times/week significantly improved physical health, mental health, and social interaction. Specifically, this frequency had a large effect on physical health (SMD = 0.64, *P* < 0.001) and social interaction (SMD = 1.02, *P* < 0.001), but had the smallest effect on mental health (SMD = 0.35, *P* < 0.001). Conversely, interventions with a frequency of < 3 times/week showed significant improvements only in social interaction (SMD = 0.16, *P* = 0.0002), with no significant effects on physical or mental health.

Due to the limited number of studies included, only two subgroups based on intervention duration were analyzed: ≥60 min and < 60 min. The classification of intervention time is based on the widely accepted belief that exercise durations exceeding 60 min can lead to muscle catabolism, resulting in a decline in testosterone levels. This may cause muscle loss rather than muscle gain or fat reduction. Considering the characteristics of the intervention time in the included studies, this analysis used 60 min as the cutoff for intervention duration. The results showed that an intervention duration of ≥60 min had significant effects on improving physical health, mental health, and social interaction among older adults. Specifically, this duration had a large effect on physical health (SMD = 0.42, *P* = 0.005) and social interaction (SMD = 0.67, *P* = 0.01), though the effect on mental health was the smallest (SMD = 0.24, *P* = 0.002). Conversely, an intervention duration of < 60 min significantly improved mental health (SMD = 0.76, *P* < 0.001) and social interaction (SMD = 0.75, *P* = 0.05), but had no significant effect on physical health.

Due to the limited number of included studies, the intervention period in this analysis was categorized into two subgroups: >12 weeks and ≤ 12 weeks. A 12-week intervention period is generally considered sufficient for exercise programs to produce significant physical and psychological effects. Previous studies have shown that short-term interventions may not allow enough time to observe sustained changes, while a 12-week period ensures that the physiological adaptations and health benefits of exercise interventions are both noticeable and maintainable. The results indicated that interventions lasting more than 12 weeks produced significant improvements in physical health, mental health, and social interaction among older adults. Specifically, the effect size for physical health was large (SMD = 0.64, *P* = 0.005), as was the effect on social interaction (SMD = 0.67, *P* = 0.01). However, the effect on mental health was relatively smaller (SMD = 0.26, *P* = 0.01). In contrast, interventions lasting 12 weeks produced significant improvements in mental health (SMD = 0.38, *P* < 0.001) and social interaction (SMD = 1.02, *P* = 0.0002), but no significant effect was found on physical health.

### Discussion sensitivity analysis and publication bias

**Figure 6:**
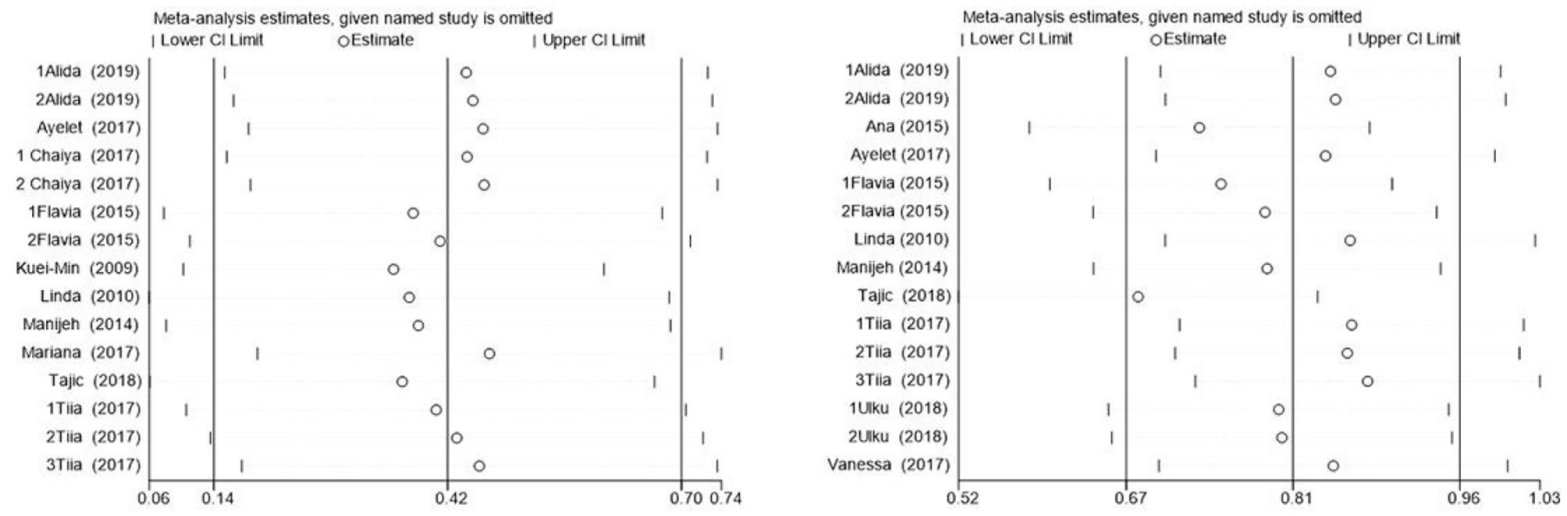
Sensitivity analysis of physical health **(left)** and social interaction **(right)**.

**Figure 7:**
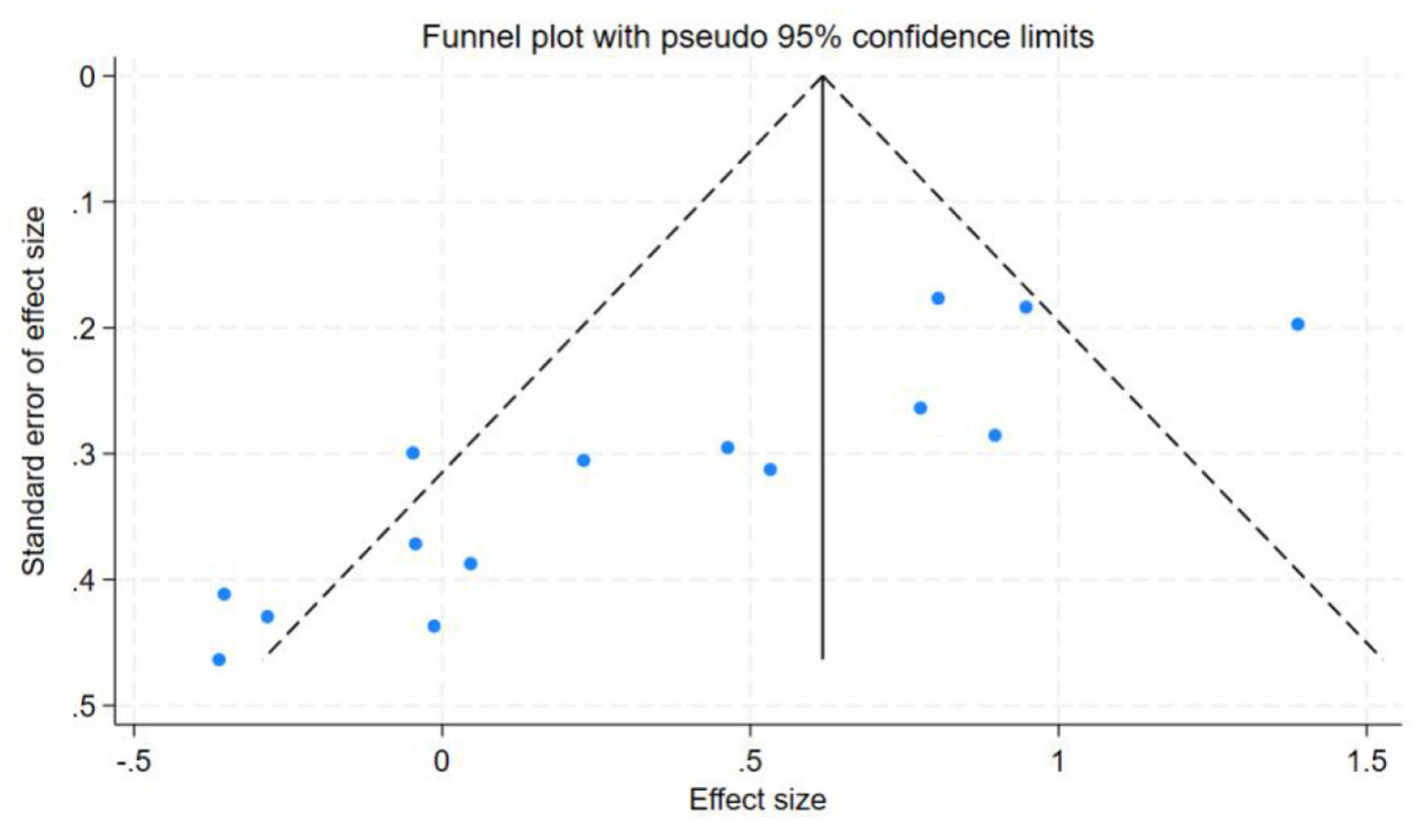
Funnel chart analysis of physical health.

**Figure 8:**
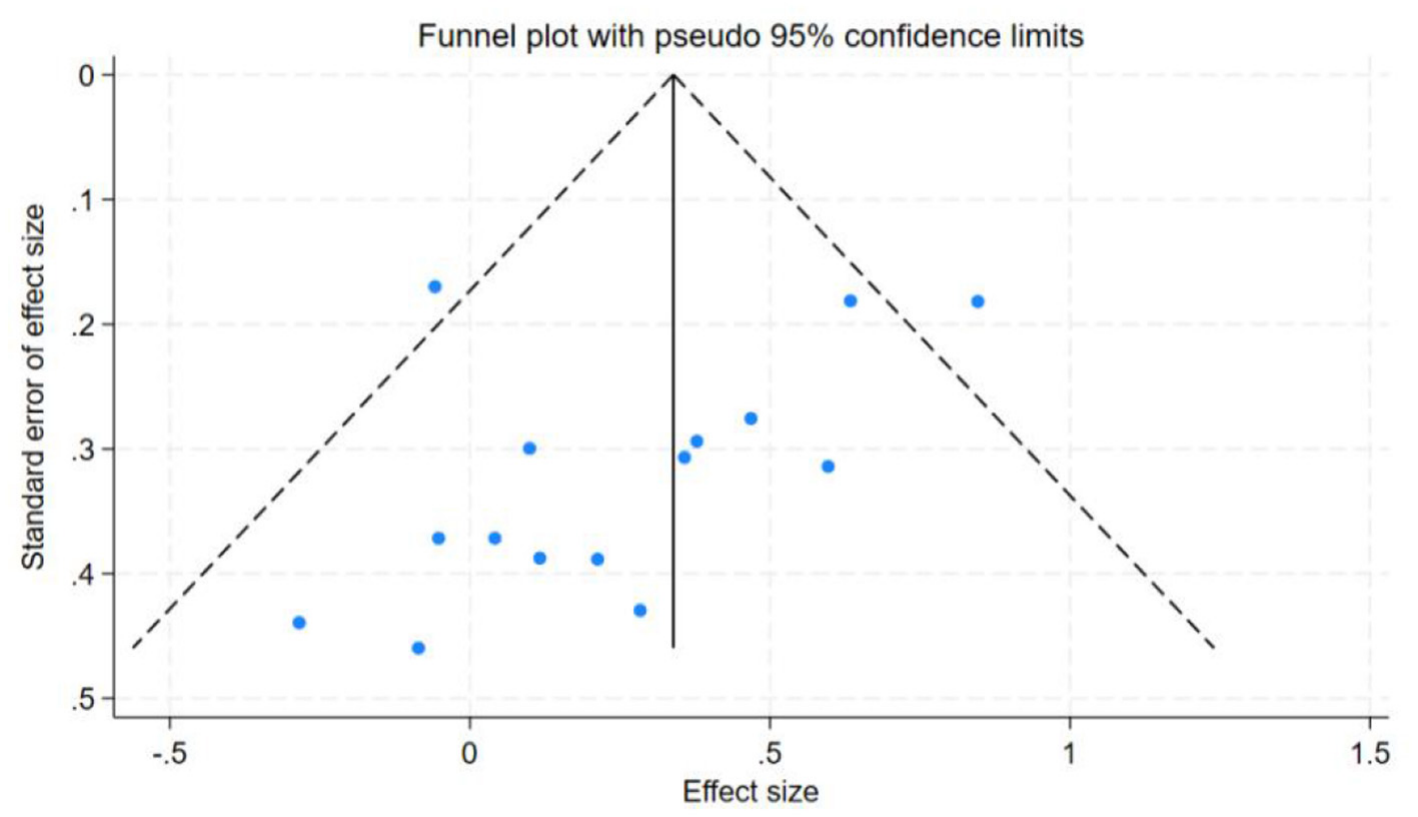
Funnel chart analysis of mental health.

**Figure 9:**
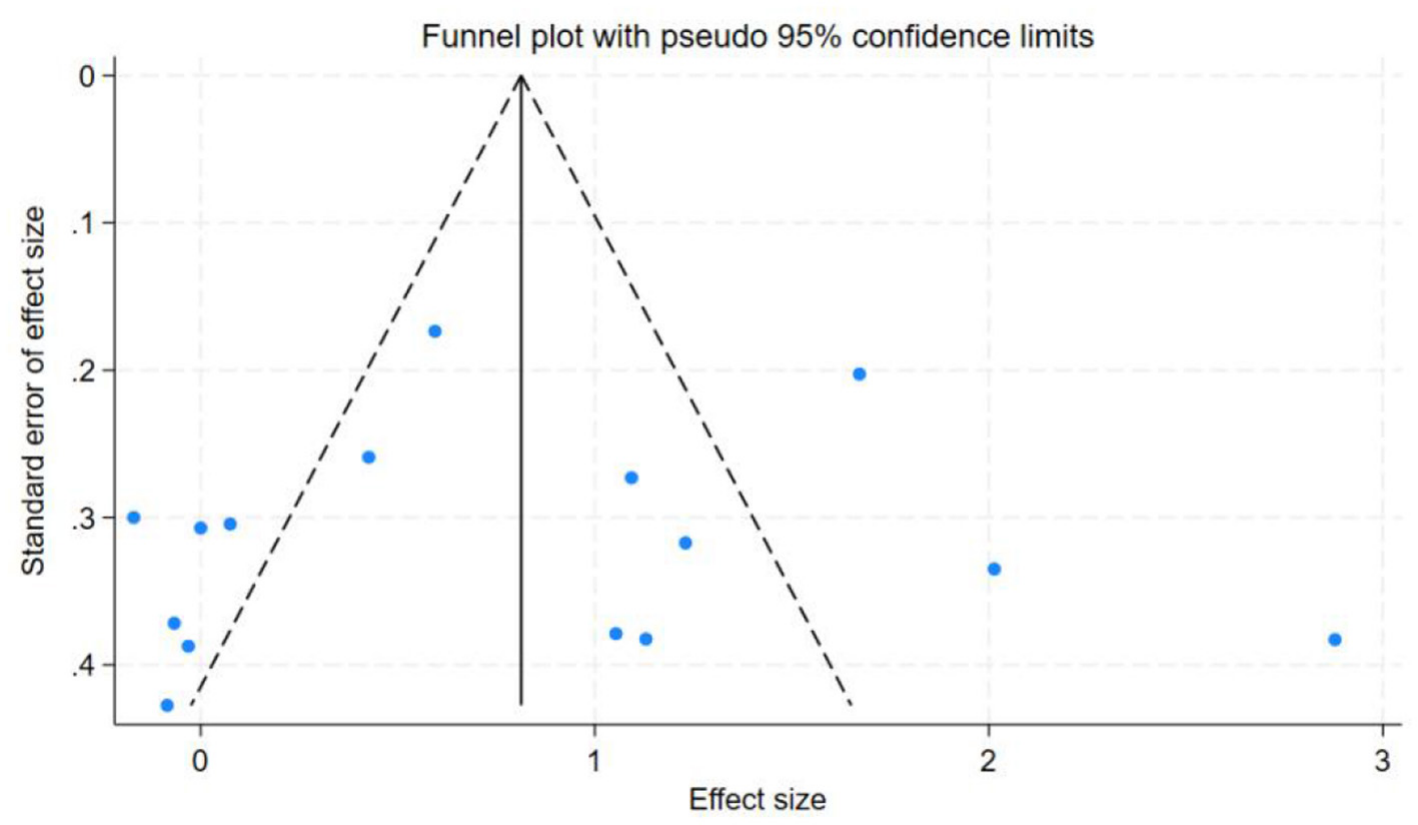
Funnel chart analysis of social interaction.

In this study, the benefits of physical exercise were examined across three dimensions: physical health, mental health, and social communication. Following heterogeneity analysis of the included studies across these dimensions, it was found that physical health (*I*^2^ = 71%, *P* < 0.001) and social interaction (*I*^2^ = 86%, *P* < 0.001) exhibited high heterogeneity, whereas mental health (*I*^2^ = 37%, *P* < 0.001) demonstrated low heterogeneity, as shown in Table 5. Therefore, sensitivity analyses were primarily conducted for physical health and social interaction, the sensitivity analysis is shown in Figure 6. Additionally, funnel plots were generated to further investigate the potential sources of heterogeneity, funnel chart analysis is shown in Figures 7–9.

The research findings indicated that a relatively high level of heterogeneity existed between physical health and social interaction. To explore and analyze this highly heterogeneous situation, we employed the Trim-and-Fill method. For physical health (*I*^2^ = 71%, *P* < 0.001), sensitivity analysis revealed that removing individual studies one by one showed that the studies by Chen et al. (27) and Tajik et al. (20) had a substantial impact on heterogeneity. After excluding Chen et al. (27), the heterogeneity decreased from *I*^2^ = 71% to 57%. Further exclusion of Tajik et al. (20) reduced heterogeneity to 50%. These findings suggest that these two studies were the primary contributors to the high heterogeneity in the physical health dimension. However, for social interaction (*I*^2^ = 86%, *P* < 0.001), sensitivity analysis indicated that removing individual studies had no significant impact on overall heterogeneity, suggesting that no single study was the primary source of heterogeneity and that the combined effect size of the study was relatively stable. However, subgroup analyses of social interaction revealed that heterogeneity remained high across the frequency, time, and cycle subgroups. This suggests that these factors may be the primary contributors to the high heterogeneity observed in the social interaction dimension. Concerning mental health, the heterogeneity was only 37%, which was categorized as low heterogeneity. Therefore, the Trim-and-Fill analysis was not utilized for the sensitivity analysis in this regard.

Publication bias tests were conducted for the studies included in the three dimensions examined in this analysis. To ensure the accuracy of the results, both the Egger test and the Begg test were performed, as shown in Table 7. The results indicated that for mental health, the Egger test yielded *t* = −1.96, *P* = 0.0712, and the Begg test yielded *z* = −1.88, *P* = 0.0748. For social interaction, the Egger test yielded *t* = 0.13, *P* = 0.8962, and the Begg test yielded *z* = 0.20, *P* = 0.8431. These findings suggest no evidence of publication bias in the mental health and social interaction dimensions. However, for physical health, the Egger test yielded *t* = −5.38, *P* = 0.0001, and the Begg test yielded *z* = −3.27, *P* = 0.0015, indicating the presence of some publication bias in this dimension.

**Table 7:** Egger test and Begg test.

**Indicator**	***t***	**Egger test**	***z***	**Begg test**
Physical health	−5.38	0.0001	−3.27	0.0015
Mental health	−1.96	0.0712	−1.88	0.0748
Social interaction	0.13	0.8962	0.20	0.8431

### Subgroup analysis

Through meta-analysis of experimental data on physical exercise and its effects on the physical and mental health of older adults, it was found that physical exercise significantly improves both physical and mental health. In terms of specific dimensions, physical exercise had the largest effect size on improving social communication, followed by a moderate effect size for improving physical health. The effect size for mental health improvement was relatively small.

Subgroup analysis of intervention methods revealed that Reed et al. (28) demonstrated that short-term aerobic exercise significantly improves physical health, particularly by enhancing cardiovascular function and muscle strength. Additionally, Reed et al. (29) found that long-term aerobic exercise significantly enhances positive emotions, especially by increasing vitality and reducing negative emotions (29). However, neither study included resistance training for comparative analysis, which limited the exploration of the different health effects of these two exercise modalities. In contrast, Ferrer et al. (30) reported through meta-analysis that moderate-intensity resistance training significantly improves social communication and quality of life in cancer patients, suggesting its potential in enhancing mental health and social function. Rodrigo et al. (31) also found that resistance training not only improves physical function in older adults but also significantly enhances their quality of life, providing strong evidence for the application of resistance training in the older adults population.

In a subgroup analysis of physical exercise dosages, Rodrigo et al. (31) suggested that resistance training with a duration of 50–70 min, performed three times per week, is necessary to improve physical function and quality of life in older adults women. Wang et al. (32) conducted a meta-analysis and found that a physical exercise intervention lasting up to 12 weeks was most effective in improving the mental health of older adults (32). Reed et al. (29) recommended at least 10–12 weeks of physical exercise, with each session lasting 30–35 min and conducted 3–5 times per week, as the optimal approach to enhance mental health. This meta-analysis found that, for improving physical health in older adults, the most effective intervention involved a cycle of at least 12 weeks, with a frequency of at least three times per week, and each session lasting no more than 60 min of low-to-moderate intensity exercise. In terms of improving mental health, the ideal intervention involved an exercise cycle of up to 12 weeks, at least three sessions per week, and moderate-intensity exercise lasting no more than 60 min per session. For improving social interaction, the recommended exercise cycle was also within 12 weeks, at least three times per week, with moderate-intensity exercise sessions not exceeding 60 min.

Subgroup analysis of intervention intensity revealed that, compared to high-intensity physical exercise, low-to-moderate intensity exercise had the most significant effect on improving physical health, mental health, and social interaction among older adults. Previous research by Reed et al. (28) and Reed et al. (29) demonstrated that low-to-moderate intensity strength training significantly improved mental health in older adults, notably reducing symptoms of depression, anxiety, stress, and other negative emotions. From a physiological perspective, most studies agree that aging leads to a gradual decline in physical function, including muscle strength, flexibility, and cardiorespiratory function (33, 34). Moreover, You et al. (35) suggested that high-intensity exercise may impose excessive strain on older adults' bodies, potentially leading to injuries, as aging bones become more fragile and prone to fractures. Psychologically, Qingwen et al. (36) found that older adults often face anxiety and stress due to aging. High-intensity exercise can exacerbate feelings of fatigue and frustration, which can negatively affect both physical and mental health. Social factors also play a significant role in determining the types of exercise older adults prefer. Economically, costs associated with fitness classes, equipment, or transportation to exercise venues can be prohibitive for low-income individuals, exacerbating disparities in access. Research by Lee et al. (16) and Tajik et al. (20) shows that as people age, their social circles tend to shrink, leading most older adults to prefer group-based, relaxed, and enjoyable physical activities, such as tai chi, which was used in the intervention group of this study. Group exercises are often more attractive to the older adults, whereas high-intensity exercise may not meet these social needs and could instead contribute to feelings of loneliness and helplessness.

Studies have demonstrated that physical exercise, as a key regulatory factor influencing human health and social interaction, offers a wide range of benefits for older adults. These benefits have been explained at the molecular level in several studies. Physical exercise plays a crucial role in stimulating neurogenesis in the brain, such as through the upregulation of growth factors and brain-derived neurotrophic factor (BDNF), which promote neurogenesis and angiogenesis, thereby alleviating depressive symptoms and improving mental health in older adults (37, 38). Barker et al. (39) suggested that physical exercise stimulates neural functions in brain regions associated with anxiety and stress, which may help reduce and alleviate anxiety symptoms. For instance, physical exercise has been shown to regulate circulating endocannabinoids, producing anti-anxiety effects by modulating neurotransmitters such as dopamine. Moreover, Mirco et al. (40) demonstrated that the release of neurochemical factors, such as opioids and endocannabinoids, promotes feelings of pleasure and happiness, thus soothing mood and restoring calm Rhyner et al. (41) found that physical exercise not only improves neuroelectrophysiological indicators in older adults, mitigating the decline in physiological function, but also regulates levels of brain-derived neurotrophic factor (BDNF), enhancing and maintaining cognitive function while helping prevent cardiovascular diseases. Through the positive emotional experiences associated with physical exercise and the development of strong social relationships, older adults can increase social interaction, which collectively improves their quality of life (42, 43). Overall, physical exercise enhances both physical and mental health in older adults, promotes neuroregulation, and fosters positive emotional experiences. Additionally, physical exercise contributes to greater social engagement, enhances self-efficacy, and further improves life satisfaction and subjective well-being in older adults.

Based on the meta-analysis results, this study recommends the following guidelines for promoting the health of older adults. To enhance physical health, a physical activity program lasting at least 12 weeks, with a frequency of no less than three sessions per week and a duration of no more than 60 min per session at low-to-moderate intensity, is optimal. For improving mental health, the exercise program should also last at least 12 weeks, with a frequency of at least three sessions per week and a duration of no more than 60 min per session at moderate intensity. To improve social interactions, an exercise program lasting no more than 12 weeks, with moderate-intensity exercise performed at least three times per week and each session lasting no more than 60 min, is most effective.

## Limitations

This study identified several limitations based on a comprehensive review of the existing literature. Firstly, many included studies did not account for potential confounding variables, such as living environment and demographic factors. The lack of control for these variables may introduce bias and affect the study's results. Future empirical studies should aim to control these factors to enhance the reliability and validity of the findings. Secondly, due to the inclusion and exclusion criteria, the search period ended in 2023. The time limit for the search did not further expand the literature. Ultimately, only 13 articles (comprising 15 studies) were included, which may limit the generalizability of the results. The small sample size in some subgroup analyses could further affect the robustness of the conclusions. Future studies should include larger sample sizes and more diverse data to improve scientific rigor and accuracy. Finally, this study was limited to English-language literature, which may restrict the breadth and scope of the findings. Future research should incorporate multilingual literature and adopt a more comprehensive evaluation approach to provide a more nuanced and complete understanding of the topic.

## Conclusion

This study investigated the impact of physical activity on the physical and mental health of older adults through a meta-analysis of data from randomized controlled trials. The results indicated that physical activity significantly improved both physical and mental health. Specifically, physical activity showed large effect sizes for enhancing social interactions, moderate effect sizes for improving physical health, and small positive effect sizes for mental health. These differences were statistically significant.

## Data Availability

The original contributions presented in the study are included in the article/supplementary material, further inquiries can be directed to the corresponding author.
